# When Fantasy Feels Too Real: The Psychological Toll of Deep Media Attachment

**DOI:** 10.7759/cureus.90249

**Published:** 2025-08-16

**Authors:** Dustin Wong, Duo Lee, Sani Shabgahi, Peter Park, Jami Wang

**Affiliations:** 1 Child and Adolescent Psychiatry, Kaiser Permanente Fontana Medical Center, Fontana, USA; 2 Psychiatry, California Health Sciences University College of Osteopathic Medicine, Clovis, USA; 3 Psychiatry, UC Riverside School of Medicine, Riverside, USA; 4 Psychiatry, Texas Christian University Burnett School of Medicine, Dallas, USA; 5 Psychiatry, Kaiser Permanente Fontana Medical Center, Fontana, USA

**Keywords:** animated series, delusions, emotional dysregulation, parasocial grief, parasocial relationships, psychotherapeutic interventions, suicidal ideation

## Abstract

Parasocial relationships (PSRs) are emotional relationships developed with a media figure. These relationships may take the form of friendship, romantic attachments, or the embodiment of the persona. This case study highlights the psychological impact of parasocial attachments, focusing on a 16-year-old female who developed a strong parasocial connection with a character from a popular animated series. The patient deeply resonated with the show and its main character, as it mirrored her own personal struggles of sexual identity, body image issues, and isolation. Unlike other viewers who likely processed the character’s death as a fictional event, the patient’s reaction was significantly more intense, triggering suicidal ideation. Following an inpatient hospitalization with medication adjustments and therapeutic interventions, the patient was discharged with improved mood. Importantly, clinicians should be mindful of the therapeutic potential of media engagement for building rapport, while also recognizing the potential negative psychological influences.

## Introduction

Parasocial relationships (PSRs) are one-sided relationships in which an individual identifies with and feels a connection to a media figure, such as a celebrity, social media influencer, or characters from shows. These relationships are unidimensional, characterized by an illusion of intimacy that often serves the desire for belonging, identification, and social needs. 

The rise of social media and technology within the last few decades has significantly increased the prevalence and intensity of PSRs, especially in adolescents. Social media platforms (e.g., Twitter, TikTok, and Instagram) and entertainment platforms (e.g., YouTube, Kick, and Twitch) have provided users with unprecedented access to media figures, enabling individuals to foster a sense of intimacy and connection. For example, adolescents who interact with their favorite media figure on Twitter through retweets or responses tend to develop stronger PSRs [1]. Furthermore, streamers on entertainment platforms such as Twitch allow direct and live interaction with their viewers through donations and live chatting, which enhances the experiences of parasocial interactions [2].

Moreover, the content and frequency of media figures who are posting online can strengthen PSRs. As previously investigated, higher-quality content and more influencer interaction are associated with stronger PSRs among followers [3]. Although PSRs can impact one’s well-being in positive aspects such as promoting healthy habits, reducing stigma, and facilitating identity exploration, they can also lead to unwanted outcomes such as negative self-comparisons, unhealthy emotional dependence, and worsening mental health [4]. We present a case revolving around an animated series from a popular streaming service, where a 16-year-old female viewer experienced a strong emotional connection to the show and its characters. The perceived possibility of a main character's death led her to believe that her own life was no longer worth living.

## Case presentation

In this case, a 16-year-old Hispanic female with a past psychiatric history of obsessive-compulsive disorder (OCD) and reported attention-deficit/hyperactivity disorder (ADHD) presented on an involuntary inpatient admission for concerns of danger to self. This was her first hospitalization with no prior suicide attempts. According to her legal hold, the patient had attended a streaming premiere for a popular animated series, where a character she resonated with potentially passed away. The belief of the character dying triggered the patient’s thoughts of wanting to end her own life. The patient stated, “The world is ending … a piece of me is gone … I can kill myself.” The patient then asked a friend for a knife, which led to a call to the police, who brought her to the hospital.

When the patient was recalling the reason for her hospitalization, she recalled her symptoms starting after finishing the show. Her concerns encompassed the possible passing of one of the main characters. The patient stated that “no other person or character” had ever felt so similar to her. With the possible death of the character and the producers announcing there would not be a renewed season of the show, the patient was distraught about the end of the character’s story. In addition, the patient had a strong connection with the show because it related to the themes of body image issues, sexual identity exploration, emotional dysregulation, and isolation. 

When discussing prior trauma, the patient denied any abuse or in-person trauma history. She expressed prior experience of grief from the death of her “brother”, Spider-Man, in the movie Avengers: Endgame. The patient could discern that the actor portraying Spider-Man, Tom Holland, was not her brother, but felt the character he portrayed was. Interestingly, the patient did not make the same connection to Spider-Man played by other actors, including Tobey McGuire and Andrew Garfield. When assessing the patient’s ability to form relationships, the patient discussed a poor relationship with her biological father. The patient also discussed having an “imaginary dad” whom she would turn to during difficult times. The patient described this imaginary figure as fulfilling the father she wanted. She also expressed that she did not have close friends with whom she could enjoy discussing shows, cosplaying, or talking about stressors. 

The patient was hospitalized in the inpatient setting for six days. During this time, the patient’s home fluoxetine of 30 mg was increased to 40 mg daily. The patient tolerated the medication with no reported side effects. The patient also actively participated in group therapy and individual therapy. Moreover, the patient had an extended discussion with the psychiatry team on theories about the film character she believed had passed and the possibilities of the character still being alive. In addition, a discussion on what the characters in the show would want for her, if she were a friend or the embodiment of the character, was helpful in thinking about how to move forward. After these conversations, the patient reported an overall improvement in mood. The patient was eventually discharged with aftercare appointments.

Given the patient's ability to reality-test between actors and characters, a primary psychotic disorder was considered less likely. Instead, her symptoms appeared to be contextually driven and tied to themes of identity, loss, and emotional dysregulation, which raised concerns for a depressive disorder with borderline delusional beliefs. As a result, her medication was increased to target her depressive symptoms. Outpatient recommendations included further optimizing her antidepressant, exploring therapy to target emotional regulation and challenging some of these parasocial beliefs, and considering low-dose atypical antipsychotics if intrusive thoughts or mood instability were problematic. 

## Discussion

While PSRs are a well-documented phenomenon, the association between intense PSRs and acute mental health crises remains relatively underexplored in psychiatric literature. However, previous research has shown that individuals often identify with media personalities as a way to cope with loneliness, using identification as a defense mechanism against social alienation [5]. In addition, one study of Chinese female college students revealed strong associations between the strength of PSRs and the levels of social anxiety and relational victimization [6]. Another study found that the intensity of grief was correlated to one’s emotional connection, regardless of whether they knew someone in person (e.g., a neighbor) or through media (e.g., a celebrity). The study demonstrated that emotional connection, or how they felt about someone, determined how deeply they grieved a loss, even if there was never any in-person interaction [7]. Similarly, as seen with the series 13 Reasons Why, a study found that the show was associated with a significant increase in monthly suicide rates among American youth aged 10 to 17 years old [8]. In particular, teenagers may be more vulnerable to the effects of PSRs, as research shows that feelings of parental rejection in childhood are linked to higher social anxiety, which can heighten emotional connections to celebrities in the media [9]. These findings suggest that parasocial connections, especially in emotionally driven media, can exacerbate emotional distress and mental health struggles in vulnerable populations, such as adolescents. 

This case is unique in several respects. First, the patient’s identification with the show's character was deeply rooted in shared themes of personal struggle and feelings of isolation. Most PSRs are formed to fulfill complex psychological needs, such as emotion regulation, by offering consistent support without the risk of rejection or betrayal [10]. However, this relationship went beyond typical admiration, instead reflecting an intimate resonance with the character’s vulnerabilities, blurring the lines between fiction and reality. This distortion of reality was so strong that the patient experienced a personal death of her own when she believed the character had died. 

Second, the patient’s history of creating an "imaginary" parental figure and her selective attachment to specific portrayals of characters, such as Tom Holland’s Spider-Man, highlights a complex interplay of unmet relational needs and media-driven connections. This suggests a deeper psychological reliance on PSRs as a compensatory mechanism for real-life interpersonal deficits. The patient’s reaction to the potential death of the show's character illustrates how the loss of a parasocial figure can mirror or amplify existing emotional wounds.

Third, the treatment team felt the most impactful improvement in the patient’s mood was being able to discuss the characters and the show. Being able to speak the “language” of the patient and utilize the characters’ positive traits to apply to herself helped the patient find positive things to look forward to in life. It is important to consider leveraging parasocial connections in therapy by discussing the character’s perspective and what goals may apply to the patient, while carefully avoiding reinforcing possible delusional thoughts. This approach allowed the patient to reframe her relationship with the character in a way that fostered healing and emotional growth. 

In summary, PSRs may offer both positive and negative benefits. On one hand, PSRs can provide a sense of belonging for individuals who may feel marginalized due to factors such as race or sexual orientation, as well as during periods of social isolation, such as the COVID-19 pandemic [10,11]. On the other hand, as this case demonstrates, PSRs can negatively impact one’s mental health, potentially leading to severe consequences, including suicidal thoughts. Currently, there are no standardized treatment protocols for intense PSRs. As seen in this case, clinical management is typically adapted from existing psychotherapeutic approaches such as cognitive behavioral therapy, supportive psychotherapy, or interpersonal therapy to address an individual's specific needs. With the growing influence of PSRs, media literacy education is crucial for both consumers and their support systems. Several considerations for clinicians include exploring media influences, monitoring specific content consumption, and assessing the emotional impact on their patients. Furthermore, clinicians can explore ways of utilizing PSRs to open up discussions and promote positive psychological and emotional well-being. By teaching individuals to critically engage with media content and recognize the boundaries of PSRs, clinicians may reduce the risk of adverse emotional outcomes.

## Conclusions

Overall, PSRs are emotional relationships formed with media personas, oftentimes driven by a desire for connection and belonging. With the rise of social media and streaming platforms, the prevalence and intensity of these relationships have begun to blur the boundaries between fiction and reality, shaping the emotional and psychological landscapes of children and adolescents. Although PSRs may allow open discussion and feelings of connection for individuals, this case also highlights the potential risks related to intense parasocial attachments. The case demonstrates the importance of clinicians staying vigilant in noticing and addressing the emotional impact of media consumption before serious psychiatric issues occur. Clinicians should consider integrating media consumption assessments by asking about a patient's engagement with shows, games, and social media. Beyond the clinical setting, caretakers and educators should be empowered to guide healthy media consumption and encourage open dialogue about shows or characters. Furthermore, this raises questions about the media industry's ethical responsibility to its audience, especially concerning content aimed at emotionally vulnerable populations. Ultimately, this case serves as a powerful reminder that while media can be a source of comfort, it also requires a thoughtful and responsible approach from clinicians, caretakers, and creators alike.
